# Upper Thoracic Pyogenic Spondylitis With a Paravertebral Abscess Treated With a Combination of Cranked Minimally Invasive Spinal Instrumentation and Negative Pressure Wound Therapy: A Case Report

**DOI:** 10.7759/cureus.72455

**Published:** 2024-10-26

**Authors:** Chikara Hayakawa, Ichiro Okano, Koki Tsuchiya, Ryo Yamamura, Yoshifumi Kudo

**Affiliations:** 1 Department of Orthopaedic Surgery, Showa University, Tokyo, JPN

**Keywords:** minimally invasive spine surgery, negative pressure wound therapy, pyogenic spondylitis, spine instrumentation, thoracic spine infection

## Abstract

Pyogenic spondylitis is generally managed conservatively; however, surgical intervention may be required depending on the patient's condition. In this report, we present a case of upper thoracic pyogenic spondylitis with a paravertebral abscess that was successfully treated using negative pressure wound therapy (NPWT) combined with a cranked rod construct and a minimally invasive posterior-only approach. This report was constructed based on previous medical records and imaging findings. A 51-year-old man with a paravertebral abscess developed pyogenic spondylitis of the upper thoracic spine (T2 and T3). Surgical treatment was chosen due to severe neurological deficits and the presence of a paravertebral abscess. However, a posterior approach for drainage and debridement was implemented to avoid damage to proximal organs. Posterior instrumented fixation surgery from the cervical spine (C6) to the thoracic spine (T6) was performed to reduce pain and maintain alignment owing to the instability of the affected vertebrae. After pedicle screw insertion, open debridement and drainage of the T2/T3 disc space and paravertebral abscess were performed using a costotransversectomy window. Because debridement was only partially possible intraoperatively with the posterior approach, NPWT was used in combination with this approach to ensure continuous postoperative drainage. To avoid exposure of the metal instrumentation to the contaminated area, the right-side rod was passed around the drainage side with connectors. Two weeks after NPWT, the wound was closed under general anesthesia. Herein, we describe the successful treatment of primary pyogenic spondylitis with a paravertebral abscess in the upper thoracic spine using posterior instrumentation surgery combined with NPWT. In cases where complete debridement of infected tissue is not achieved, NPWT may serve as a valuable adjunctive treatment.

## Introduction

Pyogenic spondylitis is usually treated conservatively with antibiotics [1]. Surgical intervention may be required in cases of severe vertebral destruction, spinal abscess formation, mechanical instability, neurological deficits, or severe pain that limits daily physical activity [1,2].

Surgical options are tailored based on the patient's condition and the location of the pathological lesions. The options include posterior decompression, posterior fusion, and anterior curettage and bone grafting. The difficulty of performing the anterior approach varies depending on the surgical level. Specifically, anterior curettage in the upper thoracic spine involves the risk of injuring the organs encountered during the approach. Consequently, various complications have been reported [3]. A posterior-only approach appears to carry lower surgical risks compared to an anterior approach. However, adequately debriding infected anterior tissue can be challenging with a posterior approach, potentially resulting in recurrence and the need for multiple surgeries [4].

Negative pressure wound therapy (NPWT) is a technique used to treat chronic or infected wounds and is suitable for local infection control [5,6]. By applying negative pressure, NPWT can remove internal micro-abscesses and debris and may facilitate the healing process, and there is potential for spinal infection treatment. NPWT could be a viable option in cases with pyogenic spondylitis where primary debridement is not possible for specific reasons. However, to the best of our knowledge, there have been no reports of NPWT performed for primary pyogenic spondylitis. Moreover, when combining posterior instrumentation surgery with NPWT, there is a concern that the implants may be exposed to pathogens. In this report, we present a case of upper thoracic pyogenic spondylitis with a paravertebral abscess that was successfully treated using NPWT combined with a cranked rod construct and a minimally invasive posterior-only approach.

## Case presentation

**Figure 1:**
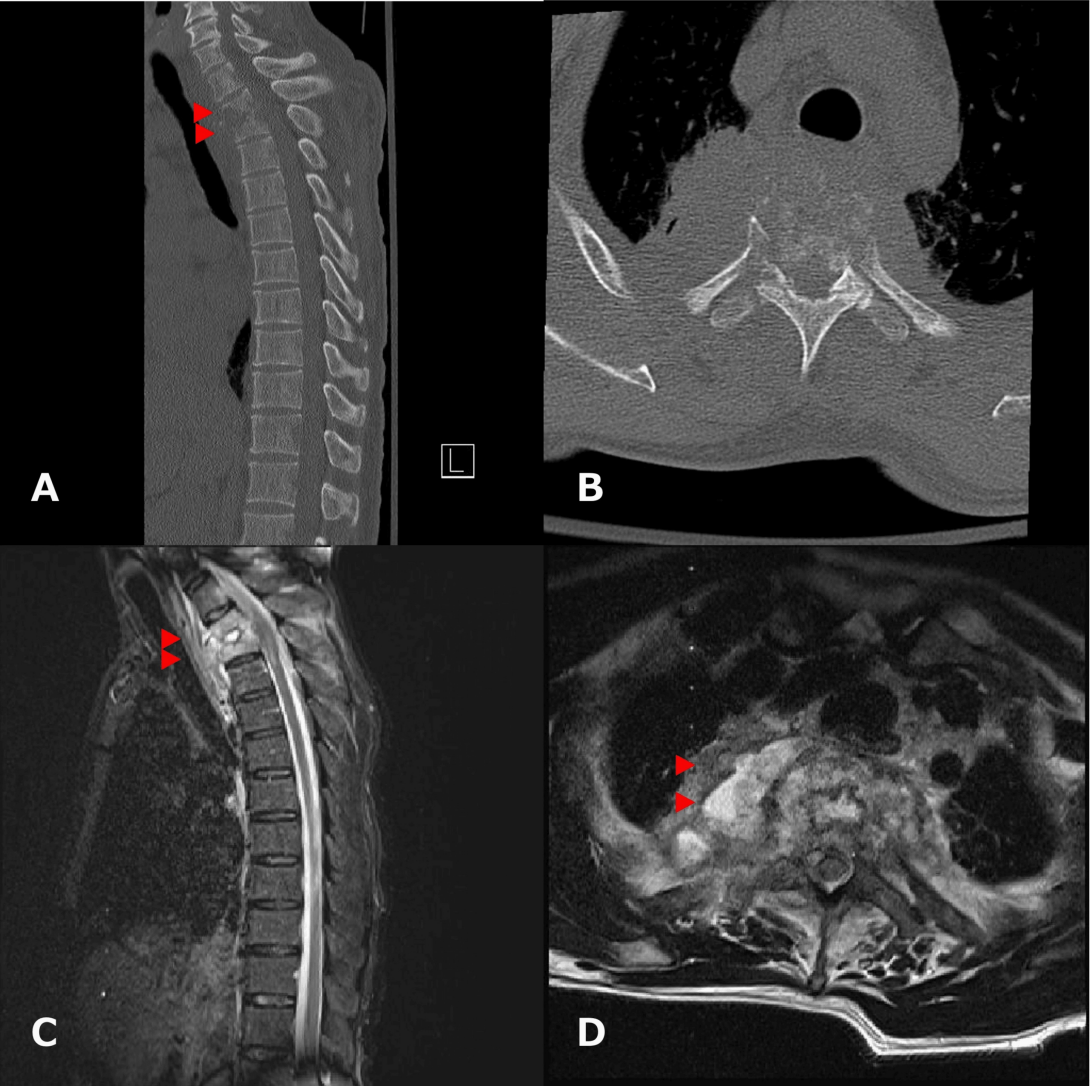
Preoperative images (A and B) Computed tomography scan showing osteolytic lesions in T2 and T3 vertebral bodies and a paravertebral soft tissue mass (arrowheads). (C and D) Magnetic resonance imaging (STIR) showing signal intensity changes in the T2 and T3 vertebral bodies with a paravertebral mass (arrowheads). STIR: short inversion time inversion recovery

A previously healthy 51-year-old male gardener presented to our hospital complaining of back pain and difficulty moving both legs. He also had numbness of the trunk and progressive paralysis of the lower extremities that appeared two weeks ago, leading to a gait disturbance. The patient also complained of gradual-onset dysuria. Physical examination revealed no neurological symptoms in the upper extremities; however, sensory deficits were observed below T5. Voluntary muscle contractions were not observed below the iliopsoas muscles. Additionally, the patient had a history of urinary incontinence. His body mass index was 21.9 kg/m^2^. The laboratory tests revealed an elevated white blood cell count and C-reactive protein (CRP) level at 13,900/µL and 17.9 mg/dL, respectively. His hemoglobin A1c level was within the normal range, and his human immunodeficiency virus test result was negative. Computed tomography (CT) of the thoracic spine revealed osteolytic lesions in the T2 and T3 vertebral bodies and a paravertebral soft tissue mass. Magnetic resonance imaging (MRI) revealed signal intensity changes in the T2 and T3 vertebral bodies and a paravertebral mass (T1, low; T2 and short inversion time inversion recovery, high), indicating thoracic pyogenic spondylitis with a paravertebral abscess (Figure 1).

The patient was subsequently admitted to our hospital. Although the patient had neurological symptoms, the spinal cord compression on MRI was minimal. Percutaneous CT-guided drainage of the paravertebral abscess was performed immediately after admission. After the microbiological workup, antibiotic therapy was initiated empirically with tazobactam/piperacillin and then de-escalated to cefazolin after the identification of the causative organism, *Staphylococcus aureus*, on the fourth day after admission.

**Figure 2:**
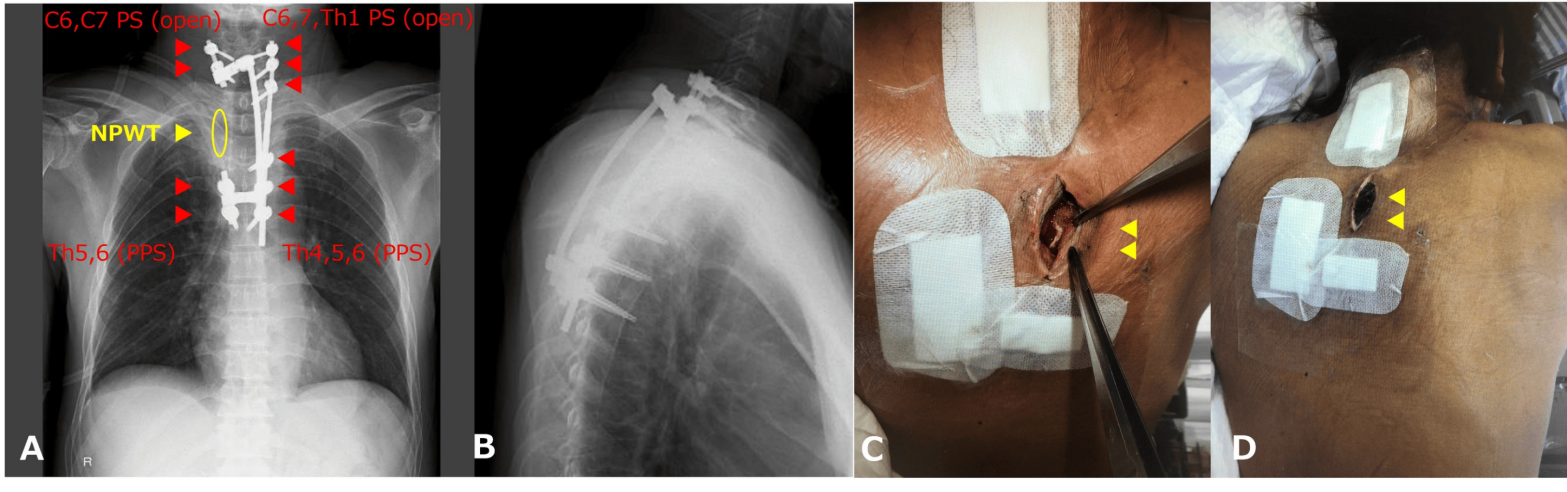
Postoperative images and clinical photographs (A and B) Postoperative anteroposterior and lateral radiographs are shown. For NPWT, a foam sponge was placed in the area circled in yellow. (C) Skin incision area with drainage (arrowheads). (D) A foam sponge for NPWT was placed on the skin incision (arrowheads). NPWT: negative pressure wound therapy

After seven days of antibiotic treatment, posterior instrumented fixation surgery from the cervical spine (C6) to the thoracic spine (T6) was performed to alleviate pain and maintain alignment due to the instability of the affected vertebrae. Pedicle screws were inserted from C6 to T1 with a mini-open approach with a 7 cm midline incision and percutaneously from T4 to T7 with skipped multiple incisions. To facilitate the debridement from the right side and minimize the risk of contamination, screws at T1 and T4 were placed only on the left side. Additionally, to avoid exposure of the metal instrumentation to the contaminated area, the right-side rod was passed around the drainage side with connectors. After all incisions were closed, an additional 4 cm longitudinal incision was made over the T2/T3 facet joint, and open debridement and drainage of the T2/T3 disc space and paravertebral abscesses were performed via a costotransversectomy window. Finally, for NPWT, a foam sponge was inserted in the wound to reach the paravertebral space, and a negative pressure of 50-75 mmHg was applied using a portable NPWT machine (3M^TM^ Acti V.A.C.^TM ^system, 3M Company, MN) (Figure 2). The foam was changed twice a week at the bedside for two weeks.

**Figure 3:**
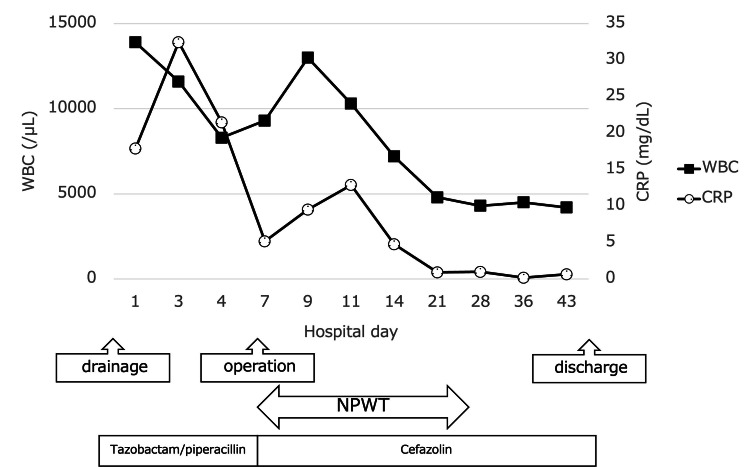
Clinical course of the patient WBC: white blood cell, CRP: C-reactive protein, NPWT: negative pressure wound therapy

After two weeks of NPWT, the wound was closed under general anesthesia. Muscle strength and sensory function in the lower extremities gradually improved. After four weeks of antibiotic treatment, his CRP level turned negative (Figure 3), his antibiotics regimen was changed to oral clindamycin for an additional six weeks, and he was transferred to a rehabilitation hospital five weeks postoperatively.

**Figure 4:**
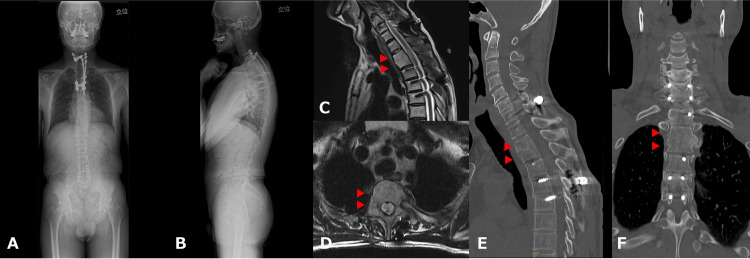
Imaging findings at one year postoperatively (A and B) Full-spine radiograph in a standing position showing good alignment maintenance. (C and D) Magnetic resonance imaging showing the disappearance of the abscess (arrowheads). (E and F) Computed tomography showing bone fusion of the affected vertebral bodies (T2 and T3) (arrowheads).

At the six-month follow-up, he was able to walk independently using a Lofstrand crutch. CT confirmed bone union, and the implant was removed 15 months after the first surgery (Figure 4).

## Discussion

In this report, we describe a case that highlights the efficacy of NPWT and cranked rod construction for treating spinal infections at the upper thoracic level. Owing to concerns regarding the risk of organ injury, we chose a posterior-only approach for drainage and debridement. Because debridement was only partially possible intraoperatively using the posterior approach, NPWT was used in combination with this approach to ensure continuous postoperative drainage.

Epidural and paravertebral abscesses may be resistant to antibiotic treatment [7]. Thus, surgery is usually considered for the treatment of these abscesses. The approach to each vertebral body varied according to the level of the affected thoracolumbar vertebrae. Historically, anterior approaches have been employed for lesions involving the vertebral body. A thoracotomy or extrapleural approach for the mid to lower thoracic spine and a retroperitoneal approach for the lumbar spine are commonly used. For the upper thoracic region, although it is rarely utilized for pyogenic spondylitis in contemporary practice, sternotomy with a longitudinal split approach and right infra-axillary thoracotomy approach could be utilized but are unfamiliar to most spine surgeons [8]. Because the choice of these approaches is based on the anatomical characteristics, the complication risk of each approach depends on the adjacent organs. Surgeons are particularly hesitant to perform longitudinal sternotomies, possibly due to the risk of significant vessel injury. Therefore, a posterior-only approach may be preferable if efficient infection control in the anterior aspect is possible. Several studies have investigated the efficacy of posterior-only approaches for spinal infections in general. Kao et al. compared the surgical outcomes of anterior-posterior combined surgery and one-stage posterior surgery with transforaminal and costotransversectomies for pyogenic spondylitis of the thoracic spine [9]. They reported that the outcomes of the posterior-only approach were not inferior to those of the combined surgery and that the posterior-only approach could be safely performed. Hadjipavlou et al. also reported the efficacy of percutaneous transpedicular discectomy for pyogenic spondylitis, but they recommended that this method be limited to patients without instability, kyphosis from bone destruction, or neurological deficits [10].

In this case, we utilized NPWT. NPWT is effective in treating infections by preventing abscess stagnation in wounds. Initially introduced to promote wound healing in trauma, NPWT is now used for diabetic foot ulcers, post-amputation wounds, and postoperative wound dehiscence in various anatomical regions [11]. Wounds can be protected from contamination and infection by closing them using drapes. The suction force from the negative pressure removes excess exudate, bacteria, and waste, effectively reducing edema and bacterial counts. It also draws in the wound edges to shrink the wound [11]. NPWT has been utilized in thoracic and abdominal surgical fields, and NPWT is safely employed when organs or vessels are exposed. [12] Furthermore, reports indicate that NPWT is not contraindicated in cases with exposed dura, provided there are no signs of cerebrospinal fluid (CSF) leakage [13]. We have been setting the NPWT pressure below 75 mmHg to prevent delayed CSF leaks or excessive bleeding. Although there is ongoing debate, studies have demonstrated that NPWT at lower pressures, around 75 mmHg, appears to be as effective as the higher setting of 125 mmHg [14]. In the field of spine surgery, NPWT is increasingly being used for postoperative infections [4], and there have been reports of cases in which infections were successfully treated while preserving metal implants [4,15]. However, there have been no reports on the use of NPWT for primary pyogenic spondylitis. NPWT has been used to treat spinal surgical site infections, especially after instrumented procedures. Certain types of bacteria, including *Staphylococcus* spp., form biofilms that improve the survival of these organisms by preventing antibiotics from reaching them when bacteria adhere to metal implants [16]. NPWT is believed to prevent biofilm formation and has advantages in the treatment of spinal infections. Conversely, other studies have demonstrated good surgical outcomes with spinal instrumentation for primary spinal infections without NPWT [17]. However, these reports stated that the reconstruction was performed after sufficient debridement. Although the use of instrumentation at infected sites should not be deemed disadvantageous, it is uncertain whether spinal instrumentation can be safely performed in cases where radical debridement is not possible. In such situations, NPWT may be helpful and can be considered as a supplemental procedure in combination with surgical debridement and instrumented reconstruction.

To prevent the risk of exposure to the pathogen, distal screws were inserted percutaneously, and the rod was connected subcutaneously in a crank shape to avoid the NPWT site. The cranked rod construct is easy to apply and requires no special implants. One concern is the stability and durability of the construct. A biomechanical study demonstrated that constructs of four rods with domino connectors showed equal strength against flexion, bending, and axial rotation compared with two-rod constructs [18]. Although the exact structure of the instrumentation used in this case was different from the abovementioned constructs, the cranked construct likely provides sufficient stability and allows NPWT application without exposing the instrumentation to contaminated wounds.

## Conclusions

We presented a case of primary pyogenic spondylitis with a paravertebral abscess in the upper thoracic spine that was successfully treated with posterior instrumentation surgery combined with NPWT. In cases of pyogenic spondylitis where adequate debridement of infected anterior tissue is challenging with a posterior approach, NPWT may help control the infection and can serve as a supplementary procedure in conjunction with surgical debridement and instrumented reconstruction.
